# Monitoring of serum and urinary biomarkers during treatment of canine visceral leishmaniasis

**DOI:** 10.14202/vetworld.2020.1620-1626

**Published:** 2020-08-18

**Authors:** Alvaro Felipe de Lima Ruy Dias, Eveline da Cruz Boa Sorte Ayres, Fernanda Harumi Maruyama, Bruna Ribeiro Gomes Monteiro, Maria Sabrina de Freitas, Arleana do Bom Parto Ferreira de Almeida, Adriane Jorge Mendonça, Valéria Régia Franco Sousa

**Affiliations:** 1 Program of Postgraduate in Veterinary Sciences, Faculty of Veterinary Medicine, Federal University of Mato Grosso, Cuiabá - MT, Brazil; 2 Veterinary Clinical Pathology Laboratory, Faculty of Veterinary Medicine, Federal University of Mato Grosso, Cuiabá - MT, Brazil

**Keywords:** clinical score, cystatin C, *Leishmania infantum*, miltefosine, NGAL

## Abstract

**Background and Aim:**

Canine visceral leishmaniasis (CanL) has a broad spectrum of changes, with kidney disease being considered the main cause of mortality. Thus, this study aimed to monitor serum and urinary biomarkers in response to two short-term treatments for CanL.

**Materials and Methods:**

Thirty dogs with CanL were equally divided into two treatment groups and treated with either miltefosine (Group M) or miltefosine plus allopurinol (Group MA); the groups were evaluated before treatment and after 28 days of treatment. Physical exams were performed and hematimetric, biochemical, and urinary parameters, including urinary biomarkers cystatin C (CisC), lipocalin-2 (NGAL), and microalbuminuria, were measured.

**Results:**

Both treatments significantly reduced clinical scores (p<0.05), but only the MA group saw a reduction in the clinical-pathological score. The serum albumin and calcium levels increased significantly in the MA and M groups (p<0.05). Proteinuria and urinary density did not decrease significantly after the treatments. With regard to the biomarkers, CisC and microalbuminuria did not have any significant changes; however, NGAL was significantly reduced in the MA group (p<0.05).

**Conclusion:**

Both pharmacotherapeutic protocols promoted clinical and clinical-pathological improvements. In addition, miltefosine plus allopurinol proved to be a safe treatment due to the lack of changes detected in the monitored renal biomarkers. The treatment with miltefosine plus allopurinol proved to be the most effective, with more pronounced beneficial effects for canines with visceral leishmaniasis.

## Introduction

Canine visceral leishmaniasis (CanL) is a ­vector-borne zoonotic disease, endemic in several ­countries, including Brazil [1]. Dogs infected by the protozoan *Leishmania infantum* are considered the main reservoir of the disease in the urban environment [2].

The affected dog presents varied clinical features, from subclinical infection to severe disease with risk of death [3,4]. Although lymphadenopathy and dermal changes are the most common clinical signs observed in this disease [2,5], renal dysfunction is frequent and is associated with increased mortality [6]. Renal involvement seems to be related to the deposition of immune complexes [4,7], leading to a decrease in the glomerular filtration rate, and consequently, can cause subclinical kidney disease, with mild and persistent proteinuria, or worsen into glomerulonephritis and interstitial nephritis [8-10].

Used as biomarkers for the diagnosis and monitoring of kidney disease, serum creatinine and urinary protein to creatinine ratios (UP/C) are recommended by the International Renal Interest Society [11] and are used to classify CanL in its various clinical stages [3]. However, they are considered suboptimal in detecting early stages of kidney disease [12]. In this context, biomarkers which allow for the early detection and location of kidney damage enable rapid and adequate therapeutic interventions and improve patient prognoses. Thus, studies with cystatin C (CisC), lipocalin-2 (NGAL), and microalbuminuria have shown that these are detected early during chronic kidney disease (CKD) diagnosis compared to seric creatinine and proteinuria, and indicate the location and severity of the kidney damage [13,14].

Therefore, due to the importance of diagnosing and monitoring renal comorbidities during the treatment of CanL, this study evaluated different serum and urinary biomarkers for glomerular and tubular dysfunction in dogs, which were naturally infected with *L. infantum* and were treated with either miltefosine or miltefosine plus allopurinol.

## Materials and Methods

### Ethical approval

All protocols were designed according to the Ethical Principles in Animal Experimentation (Brazilian College of Animal Experimentation, COBEA). This study is part of a research project approved by the Ethics Committee on the Use of Animals (CEUA/UFMT, protocol number 23108.209381/2017-44). All tutors signed an informed consent form and a term of commitment.

### Study design and clinical evaluation

During the period from June 2017 to October 2019, 30 dogs naturally infected with *L. infantum*, which were previously diagnosed at the Veterinary Hospital of the Federal University of Mato Grosso (HOVET-UFMT), were included in this study. The inclusion criteria were symptoms of CanL, any breed and sex, above 6 months of age, absence of pregnancy/lactation during treatment, polymerase chain reaction (PCR) negativity for *Ehrlichia canis*, and no previous use of leishmanicidal and leishmaniostatic drugs.

The dogs were divided into two groups: Group M consisted of 15 dogs treated with a single 2 mg/kg, daily oral dose of miltefosine (Milteforan™, Virbac) for 28 days; Group MA consisted of 15 dogs treated with miltefosine (same dose as Group M) plus a 20 mg/kg oral dose of allopurinol, twice a day, for 28 days. In this study, there was no control group to assess the placebo effect of the drugs, since the disease is progressive and leads to death if no treatment is instituted; therefore, it goes against the ethical principles of CEUA/UFMT.

The dogs were examined before treatment (D0) and after 28 days of treatment (D29). A total of 14 clinical signs (clinical score [CS]), and eight clinical-pathological variables (clinical-pathological score [CPS]) were assessed using a categorized scoring system, adapted from Miró *et al*. [15] and Bruno *et al*. [16] (Table-1).

**Table-1:** Clinical and pathological scoring system for the evaluation of dogs with canine visceral leishmaniasis (CanL).

Clinical signs	Score			

	0	1	2	3
Loss of appetite	Absent	Mild	Moderate	Severe
Weight loss	Absent	Mild	Moderate	Severe
Depression	Absent	Mild	Moderate	Severe
Lymphadenopathy	Absent	Localized	Generalized	-
Keratitis/uveitis	Absent	Mild	Moderate	Severe
Conjunctivitis/blepharitis	Absent	Mild	Moderate	Severe
Mucous membrane	Normal	Congested	Pale	White
Epistaxis	Absent	Mild	Moderate	Severe
Digestive disorders	Absent	Occasional	Recurrent	Persistent
Arthropathy	Absent	Simple	Multiple	-
Ulcers	Absent	1-2	3-5	>5
Cutaneous keratoseborrhea	Absent	Mild	Moderate	Severe
Onicogriphosis	Absent	Mild	Moderate	Severe
Polyuria/polydipsia	Absent	Mild	Moderate	Severe
Clinical-pathological signs	0	1	2	3
Platelets (×10^3^/µL)	>200	100-200	99-50	<50
Urea (mg/dL)	21-59.9	60-80	81-99	>100
Creatinine (mg/dL)	<1.4	1.4-2.8	2.9-5	>5
Total protein (g/dL)	5.5-7.5	7.6-8.4	8.5-9.5	>9.5
A/G	≥0.6	0.4-0.59	0.21-0.39	<0.2
ALT (UI/dL)	21-73	74-100	101-200	>200
UP/C	<0.2	0.2-0.5	0.51-2.0	>2
Complete blood count	0=normal, 1=anemia, 2=leukocytosis, 5=anemia + leukopenia, 7=anemia + leukocytosis			

A/G=Albumin/globulin ratio, ALT=Alanine aminotransferase, UP/C=Urinary protein/creatinine ratio

### Diagnosis

The diagnosis of CanL was confirmed by the relative antibody titers ≥1:40 in an indirect immunofluorescence antibody test (IFAT, Bio-Manguinhos^®^, Fiocruz) and with a PCR technique which used bone marrow and/or lymph node aspirates [17], as previously described by Almeida *et al*. [18].

### Samples and laboratory tests

The blood samples were collected and fractionated in tubes EDTA-coated and non-EDTA-coated tubes. The serum was separated by centrifugation at 1000× *g* for 5 min, and aliquots were stored at –80°C until serological test performance – IFAT and biochemical analyses. Urine was obtained by cystocentesis. After urinalysis, the rest was centrifuged at 1000× *g* for 10 min, and aliquots of the supernatant were stored at –80°C.

Bone marrow and lymph nodes aspirates were collected from all the animals for PCR processing, after a procedure that included trichotomy and antisepsis. The bone marrow (0.5 mL) was obtained by aspiration from the manubrium of the sternum (xiphoid process), after local anesthesia with lidocaine 2%, and placed in microtubes containing anticoagulant. The ganglionar aspirates were obtained from popliteal lymph nodes with Valeri cytoaspirator and placed in microtubes containing 250 μL of sterile saline solution. All biological samples were kept at −80°C until use.

A complete blood count was performed in an automated hematology analyzer (PocH 100iV Diff™, Roche). The urea (reference range in our laboratory, 21.4-59.92 mg/dL), creatinine (reference range, 0.5-1.5 mg/dL), albumin (reference range, 2.6-3.3 mg/dL), phosphorus (reference range, 2.6-6.2 mg/dL), and calcium (reference range, 9-11.3 mg/dL) were performed using an automated biochemical analyzer (CM 250^®^, Wiener Lab., Argentina), with commercial kits (Wiener Lab), according to the manufacturer’s instructions.

Physical, chemical, and sediment tests were used to analyze the urine. The urine specific gravity (USG) was determined in a refractometer (reference range >1030), and the sediments were observed using an optical microscope. Urinary proteins and creatinine were evaluated to estimate proteinuria (UP/C, normal range <0.5). Microalbuminuria (reference range, 10-300 mg/L) was measured using a commercial immunoturbidimetric assay (Wiener Lab), previously calibrated with purified canine albumin (Abcam – ab119814), according to the manufacturer’s recommendations and the criteria proposed by Murgier *et al*. [19].

Urinary concentrations of CisC and NGAL were determined using a multiplex immunoassay (immunology multiplex assay) with Luminex xMAP^®^ technology (CKT1MAG-97K, Merck KGaA, Germany), according to the manufacturer’s protocol. The samples were analyzed, including internal quality controls (standards and controls), with MAGPIX^®^ software xPONENT^®^, version 4.2 equipment (Merck KGaA, Germany), using standard seven-point curves. These were created from known concentrations of CisC and NGAL, using a five-parameter logistic regression curve fitting method. The detection limits for CisC and NGAL were 0.002 ng/mL and 0.006 ng/mL, respectively.

### Statistical analysis

The statistical analyses were performed using SPSS Statistics software, version 23.0 (IBM Corp., NY, USA). All data were reported as means with standard errors. The homogeneity of the variables at time D0 was confirmed with the Mann–Whitney *U*-test. Subsequently, models of generalized estimating equations (GEEs) were developed to evaluate the effects of the treatments (intergroup: MA×M), and the interactions between time and the relevant treatments (­intragroup: MA=D0×D29, and M=D0×D29) on the variables. Multiple comparisons were performed with the Bonferroni *post hoc* test. The correlation between the investigated biomarkers and the other variables was calculated using the Spearman correlation coefficient test (ρ). The level of significance adopted was 5%.

## Results

**Figure-1:**
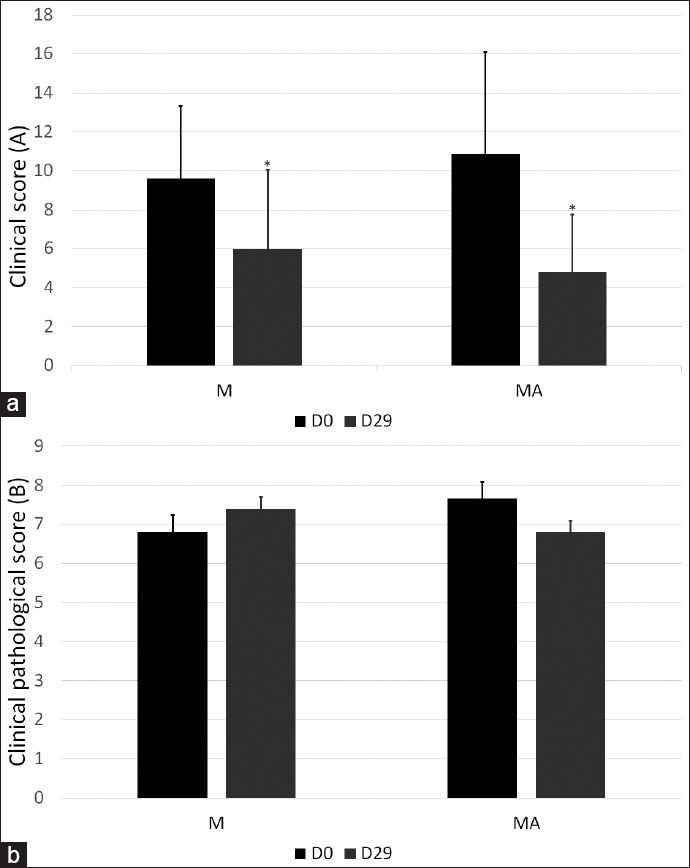
Clinical (a) and clinical-pathological (b) score of dogs treated with miltefosine (M) and miltefosine plus allopurinol (MA) during 29 days of monitoring. *p<0.05 (D0 vs. D29). p>0.05: M×MA.

During the monitoring, clinical signs improved in both groups in relation to the baselines. In the results obtained from the GEE models, a significant reduction in CS was observed in both groups at D29 (p<0.05). When the treatments were compared in terms of mean reduction in CS, there were no differences between the treatments (intergroup) (p=0.766), although the MA group obtained an average reduction of 6.06 points compared to the M group, which had an average reduction of 3.5 points (Figure-1).

Regarding CPS, no significant effects were found (p>0.05). There was a decrease in the CPS (from 7.6 to 6.8) in the MA group compared to the M group, in which the CPS increased (from 6.8 to 7.4) (Figure-1).

Serum urea and creatinine concentrations varied slightly during treatment and remained within normal limits in both groups (p>0.05). Hypoalbuminemia was found in both groups before they were treated. The mean albumin levels in relation to baseline values increased after the treatments, but they remained below the normal limit at D29. Although no intergroup differences were observed (p=0.965), the MA group showed a significant increase in albumin values at D29, demonstrating an intragroup difference (p=0.001) (Table-2).

**Table-2:** Means and standard deviations of laboratory analytes to evaluate renal function in dogs with visceral leishmaniasis, of the groups treated with miltefosine (M) and miltefosine plus allopurinol (MA), before treatment (D0) and 28 days after starting treatment (D29).

Variables	M (n=15)		MA (n=15)		p^a^ (intergroup)
	
	D0 (mean±SE)	D29 (mean±SE)	D0 (mean±SE)	D29 (mean±SE)	
Urea (mg/dL)	36.28±5.9	39.66±6.6	41.8±7.6	41.4±5.8	0.677
Creatinine (mg/dL)	0.7±0.04	0.81±0.06	0.6±0.04	0.7±0.06	0.119
Albumin (g/dL)	2.1±0.14	2.23±0.12	1.99±0.16	2.38±0.13**	0.965
Phosphorus (mg/dL)	4.8±0.43	4.38±0.3	6.2±0.31	4.7±0.34	0.058
Calcium (mg/dL)	9.34±0.36	10.94±0.22**	10.83±0.24	11.08±0.27	0.019
UP/C	2.05±0.64	1.79±0.46	1.52±0.37	1.49±0.62	0.560
USG	1037±3.7	1032±3.38	1037±3.58	1031±3.6	0.902
Cystatin C (ng/mL)	6.28±1.02	5.9±1.95	6.74±1.96	2.61±0.63	0.236
NGAL (ng/mL)	27.1±3.49	33.34±4.65	30.68±3.73	23.03±3.43*	0.487
Microalbumin (mg/L)	356.2±74.5	320.3±62.6	352±80.19	372.5±86.86	0.785

UP/C=Urinary protein/creatinine ratio, USG=Urine specific gravity.

a Treatment effect – difference between groups (M×MA).

* p<0.05 (D0×D29).

** p<0.001 (D0×D29)

The treatments promoted an increase in serum calcium concentration throughout the study, but it remained within the reference values. It was observed that the average of the differences between times D0 and D29 was statistically higher in-group M, indicating intergroup (p=0.019) and intragroup (p<0.001) differences. Serum phosphorus concentrations increased after both treatments, but no significant differences were found (p>0.05).

Proteinuria was observed in 83.3% (25/30) of the dogs before any pharmacotherapeutic interventions. In Group M, 73.3% (11/15) of the dogs had proteinuria on D0, and 80% (12/15) on D29; and in Group MA, 86.6% (13/15) of the dogs were proteinuric on D0 and 73.3% (11/15) on D29. Despite the differences, both groups showed a reduction in mean UP/C value at D29, but no significant effects were observed (p>0.05). Similarly, USG decreased in both groups, and no differences were observed (p>0.05).

Regarding biomarkers of tubular damage, CisC concentrations decreased after both treatments, with a more pronounced decrease in the MA group. However, no differences were found in the intragroup (p=0.194) and intergroup (p=0.066) analyses. Interestingly, NGAL showed contrasting results in the groups over time. The concentration of this biomarker increased in the M group and decreased in the MA group, and the MA group had a significant intragroup difference (p=0.025). However, no intergroup differences were found (p=0.487).

The glomerular damage biomarker, microalbuminuria, was above the reference values in both groups on D0, with no significant differences (p>0.05) in the groups after 29 days of treatment.

In Group M, there was a significant positive correlation between CisC and UP/C, NGAL and UP/C, microalbuminuria and UP/C, CisC and NGAL, microalbuminuria and CisC, and microalbuminuria and NGAL. A significant negative correlation was observed between UP/C and albumin, microalbuminuria and albumin, CisC and albumin, NGAL and albumin, UP/C and USG, and microalbuminuria and USG (Table-3). Meanwhile, in the MA group, there was a significant positive correlation between microalbuminuria and UP/C. A significant negative correlation was observed between UP/C and albumin, and NGAL and albumin.

**Table-3:** Correlation between biomarkers and clinical and clinical-pathological scores of the groups treated with miltefosine (M) and miltefosine plus allopurinol (MA).

Variables	Creatinine	Albumin	USG	UP/C	Cystatin C	NGAL	Microalbumin
Group M							
Creatinine		0.22	‒0.08	0.03	‒0.32	‒0.21	‒0.11
Albumin	0.22		0.03	‒0.61**	‒0.47*	‒0.55**	‒0.55*
USG	‒0.08	0.03		‒0.36*	‒0.05	‒0.06	‒0.44*
UP/C	0.03	‒0.61**	‒0.36*		0.66**	0.56**	0.67**
Cystatin C	‒0.32	‒0.47*	‒0.05	0.66**		0.47*	0.49*
NGAL	‒0.21	‒0.55**	‒0.06	0.56**	0.47*		0.55*
Microalbumin	‒0.11	‒0.55*	‒0.44*	0.67**	0.49*	0.55*	
Group MA							
Creatinine		0.29	‒0.17	0.07	0.13	‒0.08	‒0.14
Albumin	0.29		0.11	‒0.4*	‒0.11	‒0.59**	‒0.32
USG	‒0.17	0.11		‒0.2	0.02	‒0.22	‒0.19
UP/C	0.07	‒0.4*	‒0.2		0.28	0.47	0.63**
Cystatin C	0.13	‒0.11	0.02	0.28		0.21	0.02*
NGAL	‒0.08	‒0.59**	‒0.22	0.47	0.21		0.32
Microalbumin	‒0.14	‒0.32	‒0.19	0.63**	0.02	0.32	

USG=Urine specific gravity, UP/C=Urinary protein/creatinine ratio, NGAL=Lipocalin-2.

* p<0.05.

** p≤0.001

## Discussion

Dogs treated with miltefosine plus allopurinol showed a more marked recovery in CS and CPS than the dogs treated with the only miltefosine, although there was no statistical difference between the treatments. These results corroborate with the findings by Manna *et al*. [20], who considered this therapeutic protocol to be as efficient as the method of choice (antimoniate plus allopurinol), which is able to reduce CanL recurrence and promote a clinical cure within 90 days after starting treatment. It is interesting to note that in both groups, CPS recovery was slower than CS recovery. This observation can be associated with the chronic and multisystemic nature of the disease [3], which consequently delays the recovery of laboratory parameters in the short term.

Kidney damage occurs frequently in dogs with symptomatic CanL and has an occurrence which ranges from 50 to 100% [21-23]; it was observed in 25 of the 30 dogs treated in this study. Kidney disease is often associated with glomerular damage due to the deposition of immune complexes, and there is a progressive reduction in perfusion of the peritubular capillaries, leading to tubular and interstitial damage [23-25]. The progression of CKD is attributed to the overactivity of the remaining nephrons, due to the loss of glomerular units and the adaptive mechanisms, eventually resulting in glomerulosclerosis and proteinuria [8,26].

The fact that CanL results in kidney disease in the more chronic stages makes it essential to choose non-nephrotoxic drugs during CanL treatment. Meglumine antimoniate therapies can induce kidney damage, even when used temporarily [26,27]. The alternative protocol is the combination of miltefosine plus allopurinol, which is considered safe because it has a hepatic metabolism. However, self-limiting gastrointestinal disorders such as vomiting and diarrhea have been reported after miltefosine administration [28]. As in the previous studies by Nogueira *et al*. [29] and Manna *et al*. [20], we did not observe adverse effects from using miltefosine as a monotherapy (the only protocol authorized for the treatment of CanL in Brazil; Joint Technical Note No. 001/2016 MAPA/MS) or in combination with allopurinol, reaffirming its relative safety in the treatment of CanL. With regard to allopurinol, its main side effect is xanthine urolithiasis, but during a 6-year follow-up of dogs treated with allopurinol, no kidney damage was observed [20].

In this study, the dogs did not present with azotemia in either of the groups at D0 and D29. There is a consensus in the literature that seric creatinine and urea are considered markers of low sensitivity to detect kidney disease early [12], thus the changes in serum concentrations of these analytes mainly reflect the filtration capacity and not the injury markers. Urinary biomarkers of proteinuria [30] such as CisC and NGAL are more sensitive and specific in detecting glomerular and tubular damage [14], and they represent the best option for renal evaluation. This is because the use of serum biomarkers can be influenced by extrarenal factors, such as hypovolemia or urinary obstruction. Nevertheless, compensatory glomerular hyperfiltration can perpetuate serum renal markers at normal values, even when the nephrons are damaged [31].

The majority of the dogs (83.3%) had proteinuria, one of the first signs of renal impairments in dogs [26,32]. Dogs in both groups showed decreased proteinuria, suggesting that miltefosine alone or in combination with allopurinol did not impair renal function. Similarly, Bianciardi *et al*. [27] and Proverbio *et al*. [33] reported a reduction in proteinuria in dogs treated with miltefosine and miltefosine plus allopurinol, respectively. However, unlike these studies, the present study showed no significant difference in proteinuria between D0 and D29 of the pharmacotherapeutic protocols.

The concentrations of microalbuminuria were above the reference values in the evaluated dogs, and they remained so during the monitoring, although a slight decrease and increase was observed in the M and MA groups, respectively. An increase in microalbuminuria has been detected in dogs without ­azotemia, suggesting that this biomarker is an early indicator of kidney disease, compared to creatinine and serum urea. Although microalbuminuria is able to precede proteinuria in dogs [34], the stages of proteinuric kidney disease seen in 25 of the evaluated dogs did not allow for this observation.

The CisC values tended to decrease during both treatments, according to the remission of clinical signs and reduction of UP/C. However, CisC concentrations increased before the CKD azotemic stages, and it showed a more pronounced decline than serum urea and creatinine after treatments, allowing for early monitoring of renal improvements. CisC is freely filtered through the glomerulus and it is reabsorbed in the proximal tubules by endocytosis due to its size (13 kDa), eventually completely catabolized [35,36]. Therefore, our results differ from those observed by García-Martínez *et al*. [37], who noted an increase in CisC only in the azotemic stages of CKD.

NGAL values decreased significantly in the MA group at D29, while in the M group, it showed a non-significant increase, suggesting that the miltefosine plus allopurinol can contribute to a faster and more efficient reduction of renal injuries. This protein (25 kDa) is derived from neutrophils, whose increase in the urine is an indicator of renal tubular injury [14]. It is considered one of the main biomarkers of acute kidney injury (AKI) and it has been associated with AKI in dogs, with increases detected earlier than serum creatinine [38]. In CKD, significantly higher concentrations of NGAL in the urine [39] were associated with a high mortality rate. However, as this is the first study in which NGAL is monitored in dogs being treated for CanL, the lack of studies with reference values makes any comparison difficult.

The main limitation of this study was the follow-up time for the dogs, where after the 28^th^ day, for ethical reasons, all the dogs started other treatments (immunomodulators and nutraceuticals, among others). This prevented us from isolating the effects of miltefosine and/or allopurinol.

## Conclusion

After treating dogs for CanL with miltefosine plus allopurinol, there were no significant changes in serum and urinary biomarkers which detect glomerular and tubular dysfunctions. This was observed despite the pre-existing renal impairments, which were mainly marked by the occurrence of proteinuria. The treatments proved to be safe and promoted an improvement in the dogs’ clinical and clinical-pathological scores, with a more marked improvement in the group treated with miltefosine plus allopurinol.

## Authors’ Contributions

AFLRD and VRFS contributed to conception and design of the study. AFLRD wrote this manuscript. AFLRD, ECBSA, BRGM, and MSF contributed to data collection. AFLRD, FHM, ABPFA, and AJM performed analytical work. AFLRD, FRM, and VRFS critically revised the manuscript. All authors read and approved the final manuscript.

## Competing Interests

The authors declare that they have no competing interests.

## Publisher’s Note

Veterinary World remains neutral with regard to jurisdictional claims in published institutional affiliation.
